# The number of stimuli required to reliably assess corticomotor excitability and primary motor cortical representations using transcranial magnetic stimulation (TMS): a systematic review and meta-analysis

**DOI:** 10.1186/s13643-017-0440-8

**Published:** 2017-03-06

**Authors:** Rocco Cavaleri, Siobhan M. Schabrun, Lucy S. Chipchase

**Affiliations:** Brain Rehabilitation and Neuroplasticity Unit, School of Science and Health, Western Sydney University, Sydney, NSW 2560 Australia

**Keywords:** Transcranial magnetic stimulation, Reliability, Motor cortex, Cortical reorganisation, Systematic review

## Abstract

**Background:**

Transcranial magnetic stimulation (TMS) is a non-invasive means by which to assess the structure and function of the central nervous system. Current practices involve the administration of multiple stimuli over target areas of a participant’s scalp. Decreasing the number of stimuli delivered during TMS assessments would improve time efficiency and decrease participant demand. However, doing so may also compromise the within- or between-session reliability of the technique. The aim of this review was therefore to determine the minimum number of TMS stimuli required to reliably measure (i) corticomotor excitability of a target muscle at a single cranial site and (ii) topography of the primary motor cortical representation of a target muscle across multiple cranial sites.

**Methods:**

Database searches were performed to identify diagnostic reliability studies published before May 2015. Two independent reviewers extracted data from studies employing single-pulse TMS to measure (i) the corticomotor excitability at a single cranial site or (ii) the topographic cortical organisation of a target muscle across a number of cranial sites. Outcome measures included motor evoked potential amplitude, map volume, number of active map sites and location of the map centre of gravity.

**Results:**

Only studies comparing the reliability of varying numbers of stimuli delivered to a single cranial site were identified. Five was the lowest number of stimuli that could be delivered to produce excellent within-session motor evoked potential (MEP) amplitude reliability (intraclass correlation coefficient (ICC) = 0.92, 95% CI 0.87 to 0.95). Ten stimuli were required to achieve consistent between-session MEP amplitudes among healthy participants (ICC = 0.89, 95% CI 0.76 to 0.95). However, between-session reliability was influenced by participant characteristics, intersession intervals and target musculature.

**Conclusions:**

Further exploration of the reliability of multi-site TMS mapping is required. Five stimuli produce reliable MEP recordings during single-site TMS investigations involving one session. For single-site analyses involving multiple sessions, ten stimuli are recommended when investigating corticomotor excitability in healthy participants or the upper limb musculature. However, greater numbers of stimuli may be required for clinical populations or assessments involving the lower limb.

**Systematic review registration:**

PROSPERO CRD42015024579

**Electronic supplementary material:**

The online version of this article (doi:10.1186/s13643-017-0440-8) contains supplementary material, which is available to authorized users.

## Background

Transcranial magnetic stimulation (TMS) is a non-invasive means by which to assess the structure and function of the central nervous system. During TMS, a stimulation coil introduces a magnetic field over the target area of a participant’s scalp, inducing a secondary electrical current within cortical tissue [1]. Using electromyography, the muscular activation resulting from motor cortex stimulation can be measured as motor evoked potentials (MEPs). The MEP data acquired during TMS provides insight into the excitability of a participant’s neuronal network and enables changes in cortex morphology to be tracked over time [1–3]. Over the last 15 years, TMS has evolved to include applications ranging from pre-surgical tumour mapping to investigating the neurophysiological effects of various rehabilitation approaches [2–5]. Current TMS practices involve the administration of multiple stimuli over target areas of a participant’s scalp [3, 6]. Stimuli are delivered as single pulses, usually with 3 to 5 s between each stimulus [3]. Depending upon the desired neurophysiological index, stimuli may either be applied at a single cranial site, or systematically over a predefined grid, in a process known as ‘mapping’ [3].

Single-site analyses typically involve the administration of ten stimuli over the optimal cranial site, or ‘hotspot’ [6, 7]. During mapping, four to five stimuli are administered to each cranial site [3, 8]. Multiple stimuli are delivered in this manner in an attempt to minimise the influence of variations in experimenter’s technique and participant’s position [9]. Further, increasing the number of stimuli delivered per site during TMS assessments may reduce the influence of inherent variations in an individual’s corticospinal activity [6–9]. However, delivering multiple stimuli in order to map the cortical representation of a particular muscle is time-consuming, restricting its use beyond the research environment [9]. Further, prolonged TMS assessments have been associated with significant increases in participant’s fatigue and discomfort [10]. This becomes particularly important when investigating neurological populations, where increased metabolic demands and poor baseline levels of endurance may limit adherence [11]. Similarly, prolonged assessments are impractical when exploring conditions involving chronic pain [9]. The importance of reducing data collection time is also evident from the observation that corticomotor excitability fluctuates with participants’ arousal and concentration, which may vacillate throughout prolonged investigations [12].

Reducing the number of stimuli delivered to each cranial site during TMS has the potential to improve the efficiency of the procedure while decreasing participants’ demand [3]. However, lowering the number of stimuli per site may also compromise the procedure’s reliability [10]. No reviews have systematically examined the reliability of outcomes obtained using varying numbers of stimuli per cranial site during TMS [10]. Thus, the aims of this systematic review were to determine the number of TMS stimuli required to reliably measure (i) the corticomotor excitability of a target muscle at a single cranial site and (ii) the topography of the primary motor cortical representation of a target muscle across multiple cranial sites.

## Methods

This systematic review was prepared in accordance with the Preferred Reporting Items for Systematic Reviews and Meta-Analyses (PRISMA) [13] (see Additional file 1) and A Measurement Tool to Assess Systematic Reviews (AMSTAR) [14]. The methods were developed using items from the Cochrane Handbook for Systematic Reviews relevant to the reporting of systematic reviews of diagnostic reliability studies [15]. The protocol for the systematic review was registered with the International Prospective Register of Systematic Reviews (PROSPERO) (registration number CRD42015024579) and has been published previously [16].

Searches were conducted in CINAHL, CENTRAL, EMBASE, MEDLINE, Neuroscience Information Framework (NIF), PEDro, PsycINFO, PubMed, Scopus and Web of Science databases from their inception to May 2015. Key words and medical subject headings (MeSH) related to ‘transcranial magnetic stimulation’, ‘cortical reorganisation’ and ‘reliability’ were used to identify relevant literature (see Additional file 2). Search strategies were developed in consultation with a librarian with systematic review expertise and customised to suit each database. Authors contacted experts in the field of TMS and searched in Google Scholar for additional studies. Searches of the International Clinical Trials Registry Platform (ICTRP) were also conducted to identify recently completed studies. The reference lists of all relevant articles were analysed to identify additional trials to be considered for inclusion.

Inclusion criteria were limited to full-text diagnostic reliability studies comparing the effect of the number of TMS stimuli on the within- or between-session reliability of either (i) corticomotor excitability at a single cranial site or (ii) the topographic cortical organisation of a target muscle across multiple cranial sites. Only studies employing single-pulse TMS were included. The number and position of targeted cranial coordinates were required to be consistent within and between sessions. Studies comparing the reliability of TMS outcomes obtained between cortical hemispheres, rather than within or between sessions using the same hemisphere, were excluded. Similarly, studies reporting only the ‘ideal’ number of stimuli at a particular site or presenting reliability predictions based upon mathematical models, rather than experimental testing, were excluded. Reliability studies that did not explore the effect of varying numbers of stimuli were also excluded. The outcome measures that were analysed included motor evoked potential amplitude, map volume, number of active map sites, location of the map centre of gravity (CoG) and distance between the centres of gravity of the target muscle and one or more neighbouring muscles.

Search results were exported to EndNote citation software (Version X4.0.2; Thomson Reuters EndNote, New York, NY, USA) for automatic duplicate removal. Any duplicates overlooked by the program were removed manually. Two independent reviewers then screened the exported articles for relevance by title and abstract. Potentially relevant papers were retrieved as full-text articles and assessed according to the eligibility criteria by these two reviewers. An additional reviewer was consulted to resolve uncertainty or disagreement regarding the eligibility of studies. Excluded studies and reasons for exclusion were then recorded. Further data extraction procedures can be found in the systematic review protocol [16].

Two tiers of methodological quality assessment were performed. First, the general experimental design of each study was appraised using a custom appraisal tool consisting of items from both the Quality Assessment of Reliability (QAREL) checklist [17] and the guidelines for assessing reliability studies developed by Bialocerkowski et al. [18]. Second, the included studies were assessed according to the TMS-specific checklist developed by Chipchase et al. [19]. The development and justification of these quality assessment tools is outlined in detail in the systematic review protocol [16].

Two reviewers independently assessed studies satisfying the eligibility criteria. For both general experimental design and TMS-specific methodology, items were scored as either present (1) or absent (0). In accordance with QAREL-based recommendations provided by Triano et al. [20], studies scoring four or less for general experimental design were deemed to be of low quality, studies scoring between five and seven were classified as moderate quality and studies scoring eight or above were classified as high quality. Any disagreements were resolved by a third reviewer.

Reliability estimates in the form of intraclass correlation coefficients (ICCs) were pooled using StatsDirect Software (version 3.0.177; StatsDirect Ltd, Cheshire, UK) [21]. The ICC is the most accurate and commonly used indication of the size and direction of association between two variables [15]. Intraclass correlation coefficients for within- and between-session reliability were interpreted using the following values: less than 0.50 = poor; 0.50 to 0.65 = moderate; 0.65 to 0.80 = good; and greater than 0.80 = excellent [15]. Confidence intervals surrounding ICCs were calculated using the StatsDirect software, and significance was set at *p* < 0.05.

A standard correlation (Hedges-Olkin) random effects model, incorporating a Fisher’s r-to-z transformation, was used during meta-analyses. This random effects model utilised as methodological heterogeneity is inevitable during manual TMS assessments [15]. The impact of heterogeneity was calculated using the *I* ^2^ statistic and interpreted as follows: 0 to 40% may be unimportant; 30 to 60% may represent moderate heterogeneity; 50 to 90% may represent substantial heterogeneity; and 75 to 100% represents considerable heterogeneity [15]. In the cases of substantial methodological or statistical heterogeneity, subgroup analyses were performed in accordance with the study protocol.

## Results

### Flow of studies

**Fig. 1:**
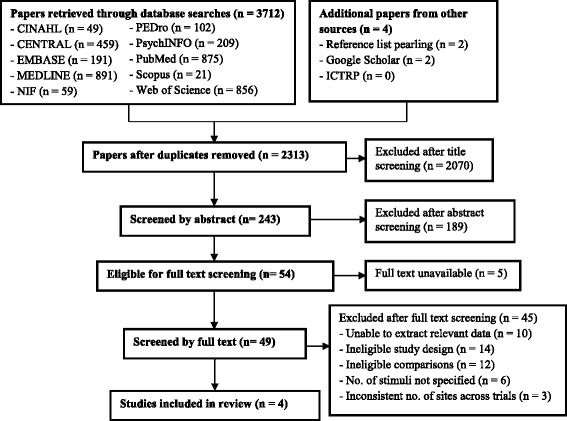
Flow of papers through review. *ICTRP* International Clinical Trials Registry Platform, *No.* number

Initial database searches yielded 3712 potentially relevant papers (see Fig. 1). Four additional studies were retrieved from other sources. No ongoing or recently completed studies were retrieved via the International Clinical Trials Registry Platform (ICTRP). Following screening, four studies met the inclusion criteria (see Fig. 1).

### Characteristics of included studies

#### Participants and assessment

Four studies contributed data on 78 participants, 51% of whom were male. The mean age of participants ranged from 27.5 to 76.0 years. Two studies investigated corticomotor excitability in the upper limb muscles [22, 23]: one study investigated the lower limb musculature [24] and one study explored the submental muscle group of the neck used during volitional swallowing [25] (see Table 1). As well as analysing data from healthy individuals, Lewis, Signal & Taylor [24] examined a clinical population in the form of individuals greater than 6 months post-cerebrovascular accident. This study was performed during active conditions, while all other included studies were performed under resting conditions. All included studies were based upon data from a single cranial site (see Table 1) and used manual (coordinate-based) coil positioning without neuronavigation.

**Table 1:** Characteristics of included studies

Study	Mean age (SD)	Sample size	M:F	Population	Investigation	TMS application	Outcome measures	Muscles tested
Bastani & Jaberzadeh [22]	30.3 (6.8)	12	6:6	Healthy	W + B session rel.	Single site	Amp	FDI, ECRB
Christie et al. [23]	76 (6.3)	30	15:15	Healthy, aged >65	W + B session rel.	Single site	Amp	ADM
Doeltgen et al. [25]	27.5 (2.9)	10	7:3	Healthy	W + B session rel.	Single site	Amp	Submental
Lewis et al. [24]	A) 57 (14) B) 55 (11.8)	26	12:14	A) 6 months post-stroke B) Healthy	W + B session rel.	Single site	Amp	Soleus

*Key*: *SD* standard deviation, *M* male, *F* female, *TMS* transcranial magnetic stimulation, *W + B* within and between, *Rel.* reliability, *Amp* amplitude, *FDI* first dorsal interosseous muscle, *ECRB* extensor carpi radialis brevis, *ADM* abductor digiti minimi

### Methodological quality

#### General experimental design

The overall experimental design quality of the studies was moderate, with a mean quality appraisal score of 6/11. Only one study was deemed to be of high quality (score ≥ 8) [22] (see Table 2). This was the only study to state that all participants received the assessment as allocated and that all participants were included in statistical analyses. None of the included studies specified participant sampling methods or performed any form of assessor blinding (see Table 2).

**Table 2:** General experimental design quality

Study	Item 1	Item 2	Item 3	Item 4	Item 5	Item 6	Item 7	Item 8	Item 9	Item 10	Item 11	Total/11
Bastani & Jaberzadeh [22]	Y	N	Y	Y	N	N	Y	Y	Y	Y	Y	8
Christie et al. [23]	Y	N	Y	Y	N	N	N	Y	Y	N	Y	6
Doeltgen et al. [25]	N	N	Y	Y	N	N	N	Y	Y	N	Y	5
Lewis et al. [24]	Y	N	Y	Y	N	N	N	Y	Y	N	Y	6

*Key*: *Y* yes, *N* no. Items: *1* clearly defined question, *2* consecutive or random sampling, *3* avoided inappropriate exclusions, *4* sample representative of target population, *5* raters blinded to additional cues that were not part of the test, *6* assessor blinding during analyses, *7* appropriate interval between tests, *8* all participants received all tests, *9* successive tests performed under same conditions, *10* all participants included in analyses, *11* appropriate statistical analyses

#### TMS-specific protocol and reporting

All studies complied with the majority of items on the TMS-specific checklist designed by Chipchase et al. [18], with scores ranging from 16 to 23 out of a possible 26. However, as demonstrated in Table 3, three studies did not report on medication use, and two did not report on the presence of participant comorbidities (including neurological/psychiatric disorders). Further, no studies controlled the level of relaxation present in the muscles other than those being tested, and only one study reported on stimulation pulse shape, current direction, interstimulus intervals or participant arousal levels (see Table 3).

**Table 3:** TMS-specific components of methodological quality

Controlled/reported	Bastani et al. [22]	Christie et al. [23]	Doeltgen et al. [25]	Lewis et al. [24]
Participant factors				
Age of subjects	Y	Y	Y	Y
Gender of subjects	Y	Y	Y	Y
Handedness of subjects	Y	Y	Y	Y
Subjects prescribed medication	N	N	Y	N
Use of CNS active drugs (e.g. anti-convulsants)	N	N	Y	Y
Presence of neurological/psychiatric disorders	N	N	Y	Y
Any medical conditions	N	N	Y	Y
History of specific repetitive motor activity	N	N	Y	N
Methodological factors				
Position and contact of EMG electrodes	Y	Y	Y	Y
Amount of contraction of target muscles	Y	Y	Y	Y
Prior motor activity of the muscle to be tested	Y	Y	Y	Y
Relaxation of muscles other than those tested	N	N	N	N
Coil type (size and geometry)	Y	Y	Y	Y
Coil orientation	Y	Y	Y	Y
Direction of induced current in the brain	Y	N	N	Y
Coil location and stability	Y	Y	Y	Y
Type of stimulator used (e.g. brand)	Y	Y	Y	Y
Stimulation intensity	Y	Y	Y	Y
Pulse shape (monophasic or biphasic)	N	N	N	Y
Determination of optimal hotspot	Y	Y	Y	Y
The time between MEP trials	N	N	N	Y
Time between days of testing	Y	Y	Y	Y
Subject attention (level of arousal) during testing	N	N	N	Y
Method for determining threshold (active/resting)	Y	Y	Y	Y
Number of MEP measures made	Y	Y	Y	Y
Method for determining MEP size during analysis	Y	Y	Y	Y
Total score/26	17	16	21	23

### Meta-analyses and subgroup analyses

Four studies compared MEP amplitudes obtained at a single cranial coordinate using varying numbers of stimuli [22–25]. No multi-site (mapping) studies satisfying the eligibility criteria were identified.

#### Within-session reliability

**Fig. 2:**
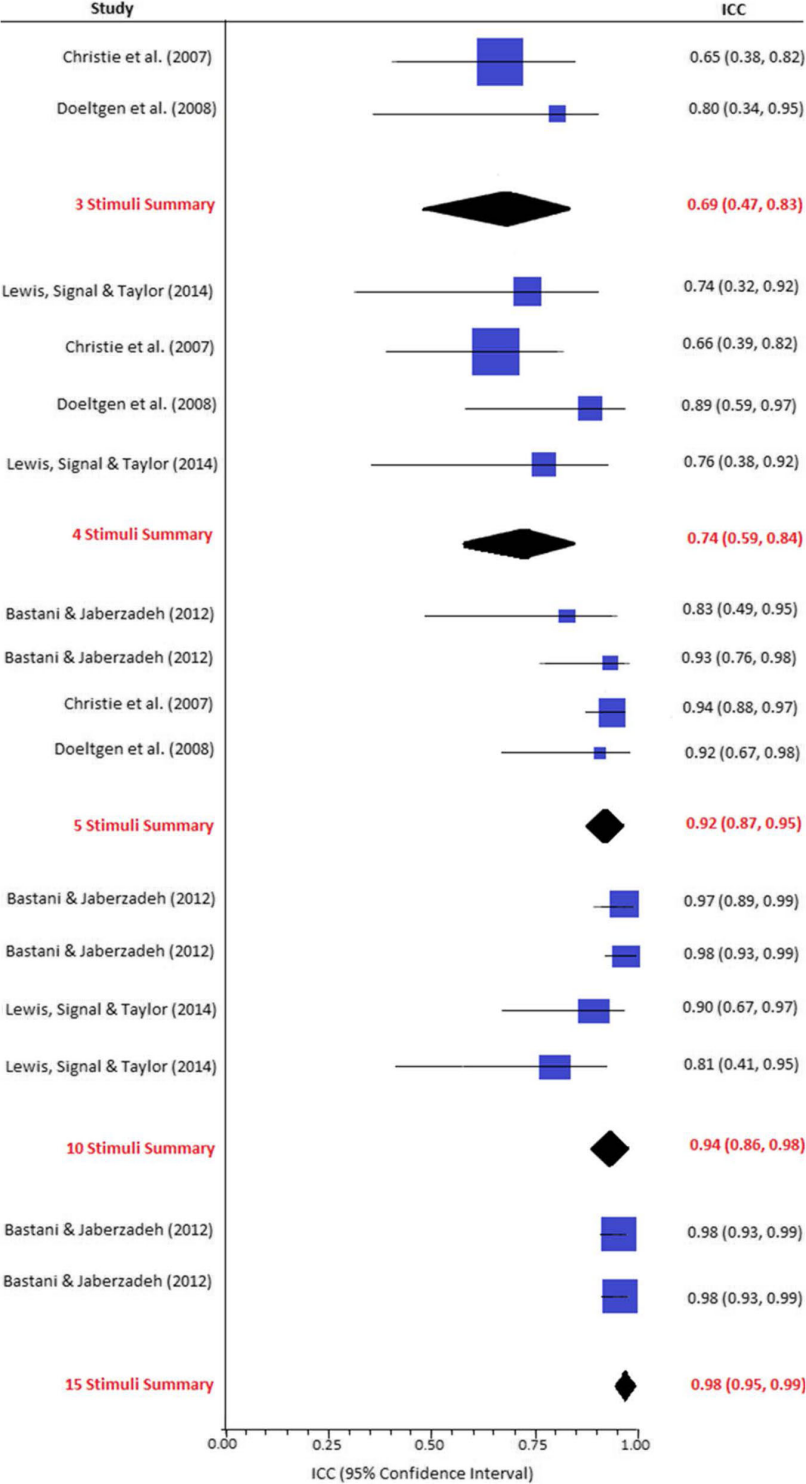
Within-session reliability of MEP amplitudes at a single site. *ICC* intraclass correlation coefficient

Four studies compared the within-session reliability of MEP amplitudes recorded using varying numbers of stimuli administered to a single cranial site [22–25]. As demonstrated in Fig. 2, the pooled ICCs for within-session reliability gradually increased from 0.69 (95% CI 0.47 to 0.83) using three stimuli to 0.98 (95% CI 0.95 to 0.99) when using 15 stimuli. Five stimuli had significantly higher ICCs than three or four stimuli (*p* < 0.01). However, no significant differences were identified between five, ten and 15 stimuli (*p* = 0.29), which all demonstrated excellent within-session reliability (ICC > 0.80). However, the analyses exhibited considerable statistical heterogeneity (ranging from *I* ^2^ = 0% [95% CI 0 to 0%]) to *I* ^2^ = 60% [95% CI 0 to 84%]). The studies also had a large degree of methodological heterogeneity in terms of target musculature and clinical population. Subgroup analyses were therefore performed to account for the potential influence of these variables (see Table 4).

**Table 4:** Subgroup analysis of within-session MEP amplitude reliability

	Pooled ICC (95% CI)					
Subgroup	*N*	3 stimuli	4 stimuli	5 stimuli	10 stimuli	15 stimuli
Healthy only	5	0.69 (0.47 to 0.83)	0.73 (0.55 to 0.85)	0.92 (0.87 to 0.95)	0.96 (0.90 to 0.98)	0.98 (0.95 to 0.99)
CVA only	1	NA	0.76 (0.38 to 0.92)	NA	0.81 (0.41 to 0.95)	NA
Age <65 years	5	0.80 (0.34 to 0.95)	0.80 (0.61 to 0.90)	0.90 (0.79 to 0.95)	0.94 (0.84 to 0.98)	0.98 (0.95 to 0.99)
Age >65 years	1	0.65 (0.38 to 0.82)	0.66 (0.39 to 0.82)	0.94 (0.88 to 0.97)	NA	NA
Upper limb only^a^	3	0.65 (0.38 to 0.82)	0.66 (0.39 to 0.82)	0.92 (0.86 to 0.96)	0.98 (0.94 to 0.99)	0.98 (0.95 to 0.99)
Lower limb only^b^	2	NA	0.75 (0.49 to 0.89)	NA	0.86 (0.68 to 0.95)	NA
Submental group only	1	0.80 (0.34 to 0.95)	0.89 (0.59 to 0.97)	0.92 (0.67 to 0.98)	NA	NA

*ICC* intraclass correlation coefficient, *CI* confidence interval, *N* number of participant cohorts, *CVA* participants with 6 months post-cerebrovascular accident, *NA* not available

^a^Also, analysis of resting conditions only

^b^Also, analysis of active conditions only

As shown in the subgroup analysis presented in Table 4, the trends observed in the overall meta-analysis remained when studies investigating healthy participants, elderly participants and the upper limb musculature were separated. This indicates that the results were not influenced by participants’ differences or methodological inconsistencies.

No statistically significant differences were observed with varying numbers of stimuli in studies investigating submental musculature or participants aged less than 65 years (see Table 4). However, in these studies, five still represented the lowest number of stimuli for which ICC confidence intervals were entirely within the ‘good to excellent’ reliability range. Intraclass correlation coefficients were also higher overall for these participant groups.

#### Between-session reliability

**Fig. 3:**
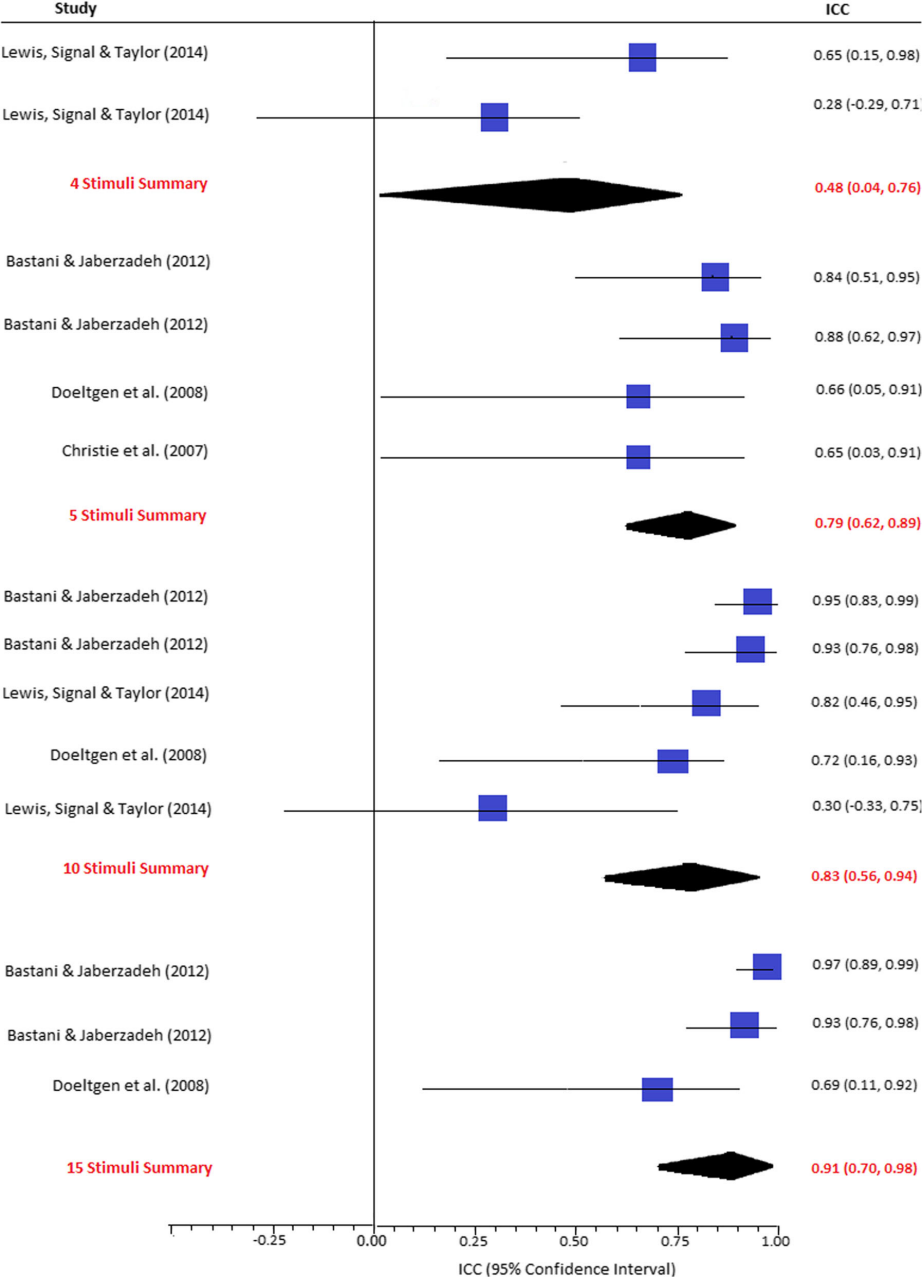
Between-session reliability of MEP amplitudes at a single site. *ICC* intraclass correlation coefficient

Four studies compared the between-session reliability of MEP amplitudes recorded using varying numbers of stimuli administered to a single cranial site [22–25]. While pooled ICCs for between-session reliability tended to increase as higher numbers of stimuli were administered (see Fig. 3), differences were not significant (*p* = 0.1). However, the analyses exhibited considerable statistical heterogeneity (ranging from *I* ^2^ = 0% [95% CI 0 to 0%] to *I* ^2^ = 69% [95% CI 0 to 86.1%]). The studies also had a large degree of methodological heterogeneity in terms of target musculature and time provided between successive sessions. Subgroup analyses were therefore performed to account for the potential influence of these variables (see Table 5).

**Table 5:** Subgroup analysis of between-session MEP amplitude reliability

	Pooled ICC (95% CI)					
Subgroup	*N*	3 stimuli	4 stimuli	5 stimuli	10 stimuli	15 stimuli
Healthy only	5	NA	0.65 (0.15 to 0.88)	0.79 (0.62 to 0.89)	0.89 (0.76 to 0.95)	0.91 (0.70 to 0.98)
CVA only	1	NA	0.28 (−0.29 to 0.71)	NA	0.30 (−0.33 to 0.75)	NA
Age <65 years	5	NA	0.48 (0.04 to 0.76)	0.82 (0.64 to 0.91)	0.83 (0.56 to 0.94)	0.91 (0.70 to 0.98)
Age >65 years	1	NA	NA	0.65 (0.03 to 0.91)	NA	NA
Upper limb only^a^	3	NA	NA	0.82 (0.64 to 0.91)	0.94 (0.76 to 0.99)	0.95 (0.89 to 0.98)
Lower limb only^b^	2	NA	0.48 (0.04 to 0.76)	NA	0.63 (−0.10 to 0.92)	NA
Submental group only	1	NA	NA	0.66 (0.05 to 0.91)	0.72 (0.16 to 0.95)	0.69 (0.11 to 0.92)
<72 h between sessions	4	NA	NA	0.79 (0.62 to 0.89)	0.90 (0.74 to 0.97)	0.91 (0.70 to 0.98)
>72 h between sessions	2	NA	0.48 (0.04 to 0.76)	NA	0.63 (−0.10 to 0.92)	NA

*ICC* intraclass correlation coefficient, *CI* confidence interval, *N* number of participant cohorts, *CVA* participants with 6 months post-cerebrovascular accident, *NA* not available

^a^Also, analysis of resting conditions only

^b^Also, analysis of active conditions only

As shown in Table 5, between-session reliability tended to increase with increasing numbers of stimuli, regardless of participant characteristics or target musculature. Overall, the ICCs for between-session reliability were significantly lower than those obtained for within-session reliability (*p* < 0.05). In healthy participants, ten stimuli were required to achieve excellent between-session reliability (ICC > 0.80). Studies involving submental musculature showed good reliability (ICC > 0.65) for all analyses, with no significant differences identified between varying numbers of stimuli. However, the results of these studies demonstrated wide confidence intervals, suggesting that higher numbers of stimuli may be required. Investigations of the upper limb musculature yielded good to excellent between-session reliability for analyses involving five, ten and 15 stimuli (see Table 5). Conversely, investigations of the lower limb musculature and individuals’ post-cerebrovascular accident (CVA) demonstrated poor between-session reliability (ICC < 0.65), even when ten stimuli were analysed. Intersession intervals less than 72 h yielded significantly higher ICCs than studies involving longer intersession intervals (*p* < 0.05) (see Table 5).

## Discussion

This review is the first to synthesise data from experimental studies comparing the within- and between-session reliability of TMS outcome measures obtained using varying numbers of stimuli per cranial site. Surprisingly, no eligible studies investigating multi-site mapping were identified. For single-site analyses, five was the lowest number of stimuli that could be delivered to produce excellent within-session MEP amplitude reliability. Increasing the number of stimuli beyond this value did not significantly increase reliability in any participant group. Conversely, between-session reliability was influenced by participant characteristics, target musculature and intersession intervals. The ICCs for between-session reliability were significantly lower than those obtained for within-session reliability, with a minimum of ten stimuli being required to achieve consistent between-session MEP amplitudes among healthy participants.

Although extensive database searches were employed, no studies investigating the reliability of varying numbers of stimuli per cranial site during multi-site TMS mapping were identified. However, single-site analyses, as explored in this review, have been used previously to inform mapping practices [26–28]. Most commonly, ten stimuli are administered over the ‘hotspot’ during single-site analyses [3]. Although this approach has been validated using magnetic resonance imaging (MRI), the International Federation of Clinical Neurophysiology highlights that current recommendations were determined arbitrarily in an attempt to ensure high levels of reliability [6]. The current review found no statistically significant differences between five and ten stimuli in terms of within-session MEP amplitude reliability, with both approaches exhibiting excellent intraclass correlation coefficients. This finding was consistent across all participant groups. Such results suggest that data acquisition times for single-site analyses involving one session may reliably be halved. Although single-site analyses are not as physiologically demanding as TMS mapping, reducing the number of stimuli required may enhance participants’ comfort. Importantly, it would also facilitate analysis of short-lived changes in corticomotor excitability, as well as exploration of the effects of interventions (such as sports taping or cortisone injections) on rapid neuroplasticity [3].

The between-session reliability of MEP amplitudes obtained following single-site TMS was also investigated. In healthy participants, ten stimuli were required to achieve excellent between-session reliability (ICC > 0.80). However, subgroup analyses revealed that this value was influenced by participant characteristics, target musculature, and intersession intervals. For example, greater numbers of stimuli were required to reliably assess participants’ post-cerebrovascular accident. Such findings are not surprising, as individuals with neurological lesions have been shown to exhibit significant variability in corticomotor excitability over time [28, 29]. Analyses of the lower limb musculature also required a greater number of stimuli to achieve high levels of between-session MEP amplitude reliability. This may be due to the difficulties associated with ensuring participants to maintain consistent submaximal contractions during TMS assessments involving the lower limb [23]. As supported by mathematical models [26], greater intersession intervals were associated with lower ICCs. The number of stimuli required during TMS assessments therefore appears to be dependent upon the period of time between sessions.

While this systematic review and meta-analysis provides researchers with useful information regarding TMS investigations, the overall methodological quality of the evidence was classified as ‘moderate’ (general experimental design score of 6/11). All studies scored poorly in the areas of blinding and sampling methodology. Participant blinding is difficult during TMS investigations, and is often not a requirement during reliability studies in which subsets of scores are compared [15, 28]. However, blinding of assessors to participant characteristics and during data analysis is usually necessary [17, 18]. Random or consecutive sampling should also be utilised during reliability studies in order to reduce the potential for bias [18]. As none of the studies employed these methodological processes, TMS procedures or sample characteristics may have been altered according to expected outcomes [15]. Additionally, only one study explicitly stated that all participants were assessed and included in statistical analyses [22]. Most of the included studies were therefore at risk of further bias associated with attrition or data exclusion [15–18].

All studies complied with the majority of items on the TMS-specific checklist [19] (scores ranging from 16 to 23 out of a possible 26). Thus, while general methodological designs may not have been robust, TMS-specific protocols were well implemented and reported. Despite this, three studies did not report on medication use [22–25], two did not report on the presence of participant comorbidities (including neurological/psychiatric disorders) [22, 23] and only one study reported on participant arousal [24]. These factors have been shown to influence corticomotor excitability and participant adherence across TMS sessions [28, 29]. Further, no studies controlled the level of relaxation present in the muscles other than those being tested, and only one study reported on stimulation pulse shape, current direction, or interstimulus intervals. This makes it difficult to definitively determine if variations in MEP recordings over time are due to true corticomotor plasticity, or simply the result of inconsistent experimental procedures [29–31].

Despite a rigorous approach towards data collection and synthesis, this review is not without limitations. As database searches were limited to full-text articles, bias may have been introduced due to the exclusion of data in grey literature. This ‘publication bias’ may inflate reliability estimates, as studies with desirable or significant results are more likely to be granted publication [15]. Additionally, including only one stimulation intensity per study (as per the review protocol) may have reduced the external validity of this review. The results of this study are therefore only applicable to the most commonly utilised stimulation intensities (110–130% of resting motor threshold). Another important limitation of the review is that it was not possible to investigate publication bias due to the limited number of articles that were retrieved for each comparison. While visual inspection of the forest plots suggests that the results were not greatly influenced by study sample size or publication date, the inability to produce funnel plots or perform formal tests of publication bias warrants further caution when interpreting the results. Similarly, this review highlighted a considerable amount of methodological and statistical heterogeneity across the included studies. Despite such limitations, the findings across the included studies were relatively consistent, even when subgroup analyses were performed. The majority of the studies were also recent, and TMS technology did not appear to vary significantly from study to study.

The findings of this review are also only applicable to ‘traditional’, systematic TMS approaches. Alternate mapping techniques, involving ‘pseudorandom’, rather than systematic, site stimulation, have shown promise in terms of improving the efficiency of TMS assessments [10]. These approaches involve delivering single stimuli to each cranial site in a pseudorandom manner, reducing the need for repeated stimulation. However, the evidence supporting such techniques is still developing, and they have only been compared to abridged TMS protocols involving the delivery of three stimuli per cranial site, rather than traditional approaches involving five stimuli per cranial site [10]. Further, the conventional systematic approach towards TMS assessment continues to be a common practice, so optimising the efficiency of this technique is an important pursuit.

While the number of studies exploring the efficiency of TMS is increasing, there remains a paucity of evidence on multi-site mapping. However, the results obtained from single-site analyses highlight the potential for reliably reducing the number of stimuli administered during TMS-based investigations. Future research should seek to determine if this potential can be translated to TMS mapping. Doing so would decrease participant demand during TMS investigations, while making data acquisition more achievable within a clinical setting and timeframe. Common indices of corticomotor excitability and organisation, including map volume, distance and number of active sites, should also be explored. The quality of future research may be improved by ensuring assessor blinding, and random sampling are performed. Studies should also utilise the TMS checklist [19] in order to provide sufficient methodological detail. This would ensure that variations in MEP recordings could more validly be attributed to changes in corticomotor excitability.

## Conclusions

This review found that delivering five stimuli produces reliable MEP recordings during single-site TMS investigations involving one session. For single-site analyses involving multiple sessions, ten stimuli are recommended when investigating the corticomotor excitability in healthy participants or the upper limb musculature. However, further studies comparing varying numbers of stimuli during TMS assessments would be beneficial, as the methodological and statistical heterogeneity observed across the included studies warrants a degree of caution when interpreting the results. Greater numbers of stimuli may also be required for clinical populations or assessments involving the lower limb. If intersession intervals exceed 72 h, reliability may be compromised. Multi-site studies, including multiple indices of corticomotor excitability and exploration of clinical populations, are required to accurately determine the optimal number of stimuli for TMS mapping.

## Additional files

Additional file 1: PRISMA 2009 checklist. (DOC 62 kb)
Additional file 2: Appendix A: Database searches. Searches were limited to full text, human studies from Jan 1980 to May 2015. (DOCX 16 kb)

## Abbreviations

AMSTAR: A Measurement Tool to Assess Systematic Reviews
CoG: Centre of gravity
ICC: Intraclass correlation coefficient
MEP: Motor evoked potential
PRISMA: Preferred Reporting Items for Systematic Reviews and Meta-Analyses
QAREL: Quality Assessment of Reliability Studies
TMS: Transcranial magnetic stimulation
