# Supplementary material for: The number of stimuli required to reliably assess corticomotor excitability and primary motor cortical representations using transcranial magnetic stimulation (TMS): a systematic review and meta-analysis
Source: Syst Rev. 2017 Mar 6;6:48. doi: 10.1186/s13643-017-0440-8 (PMC5340029; doi:10.1186/s13643-017-0440-8)
Supplement: Additional file 2: — Appendix A: Database searches. Searches were limited to full text, human studies from Jan 1980 to May 2015. (DOCX 16 kb) [file 13643_2017_440_MOESM2_ESM.docx]

**Appendix A: Database searches**

Searches were limited to Full text, human studies from Jan 1980 to May 2015

**CINAHL**

((MH "Transcranial Magnetic Stimulation") OR (MH "Magnet Therapy") OR Transcranial magnetic stimulation OR “TMS” OR magnet* ) AND ( (MH "Evoked Potentials, Motor") OR (MH "Evoked Potentials") OR MEP OR MEPs OR motor evoked potenti* OR OR CMAP OR compound motor action potential OR sites OR pulses OR stimuli) AND ( amplitude OR size OR volume OR CoG OR centre of gravity OR center of gravity OR distance OR shift OR map* OR peak OR cortical reorgani* OR excitab* OR plastic* OR neuroplast* OR cortical represent* OR (MH "Neuronal Plasticity") ) AND ( (MH "Reliability") OR (MH "Interrater Reliability") OR (MH "Reliability and Validity") OR (MH "Test-Retest Reliability") OR (MH "Intrarater Reliability") OR (MH "Equipment Reliability") OR (MH "Reproducibility of Results") OR (MH "Intraclass Correlation Coefficient") OR (MH "Internal Consistency") OR Reliab* OR reproduce* OR valid* OR consisten* OR accura* OR stable OR stability OR agree* OR “number of” OR “optimal number” OR similar*)

**PsycInfo**
( (MH "Transcranial Magnetic Stimulation") OR (MH "Magnet Therapy") OR Transcranial magnetic stimulation OR “TMS” OR magnet* ) AND ( (MH "Evoked Potentials, Motor") OR (MH "Evoked Potentials") OR MEP OR MEPs OR motor evoked potenti* OR OR CMAP OR compound motor action potential OR sites OR pulses OR stimuli) AND ( amplitude OR size OR volume OR CoG OR centre of gravity OR center of gravity OR distance OR shift OR map* OR peak OR cortical reorgani* OR excitab* OR plastic* OR neuroplast* OR cortical represent* OR (MH "Neuronal Plasticity") ) AND ( (MH "Reliability") OR (MH "Interrater Reliability") OR (MH "Reliability and Validity") OR (MH "Test-Retest Reliability") OR (MH "Intrarater Reliability") OR (MH "Equipment Reliability") OR (MH "Reproducibility of Results") OR (MH "Intraclass Correlation Coefficient") OR (MH "Internal Consistency") OR Reliab* OR reproduce* OR valid* OR consisten* OR accura* OR stable OR stability OR agree* OR “number of” OR “optimal number” OR similar* )

**PubMed**
 (((("Transcranial Magnetic Stimulation"[Mesh] OR Transcranial magnetic stimulation OR TMS OR “Magnet Therapy”[Mesh])) AND ("Evoked Potentials, Motor"[Mesh] OR MEP OR MEPs OR motor evoked potenti* OR CMAP OR compound motor action potential OR sites OR pulses OR stimuli)) AND (amplitude OR size OR volume OR CoG OR centre of gravity OR center of gravity OR distance OR shift OR map OR mapping OR maps OR peak)) AND ("Reproducibility of Results"[Mesh] OR Reliab* OR reproduce* OR valid* OR consisten* OR accura* OR stable OR stability OR agree* OR “number of” OR “optimal number” OR similar*)

**CENTRAL
ID Search**

#1 MeSH descriptor: [Transcranial Magnetic Stimulation] explode all trees

#2 MeSH descriptor: [Evoked Potentials, Motor] explode all trees

#3 MeSH descriptor: [Reproducibility of Results] explode all trees

#4 Transcranial magnetic stimulation OR “TMS” OR magnet*

#5 MEP OR MEPs OR motor evoked potenti* OR sites OR pulses OR stimuli

#6 amplitude OR size OR volume OR CoG OR centre of gravity OR center of gravity OR distance OR shift OR map* OR peak OR cortical reorgani* OR excitab* OR plastic* OR neuroplast* OR cortical represent*

#7 Reliab* OR reproduce* OR valid* OR consisten* OR accura* OR stable OR stability OR agree* OR “number of” OR “optimal number” OR similar*

#8 #1 OR #4

#9 #2 OR #5

#10 #3 OR #7

#11 #5 AND #9 AND #6 AND #10

**EMBASE
ID Search**

#1 Transcranial magnetic stimulation OR “TMS” OR magnet*

#2 MEP OR MEPs OR motor evoked potenti* OR sites OR pulses OR stimuli

#3 amplitude OR size OR volume OR CoG OR centre of gravity OR center of gravity OR distance OR shift OR map* OR peak OR cortical reorgani* OR excitab* OR plastic* OR neuroplast* OR cortical represent*

#4 Reliab* OR reproduce* OR valid* OR consisten* OR accura* OR stable OR stability OR agree* OR “number of” OR “optimal number” OR similar*

**MEDLINE**( (MH "Transcranial Magnetic Stimulation") OR (MH "Magnet Therapy") OR Transcranial magnetic stimulation OR “TMS” OR magnet* ) AND ( (MH "Evoked Potentials, Motor") OR (MH "Evoked Potentials") OR MEP OR MEPs OR motor evoked potenti* OR CMAP OR compound motor action potential OR sites OR pulses OR stimuli ) AND ( amplitude OR size OR volume OR CoG OR centre of gravity OR center of gravity OR distance OR shift OR map* OR peak OR cortical reorgani* OR excitab* OR plastic* OR neuroplast* OR cortical represent* OR (MH "Neuronal Plasticity") ) AND ( (MH "Reliability") OR (MH "Interrater Reliability") OR (MH "Reliability and Validity") OR (MH "Test-Retest Reliability") OR (MH "Intrarater Reliability") OR (MH "Equipment Reliability") OR (MH "Reproducibility of Results") OR (MH "Intraclass Correlation Coefficient") OR (MH "Internal Consistency") OR Reliab* OR reproduce* OR valid* OR consisten* OR accura* OR stable OR stability OR agree* OR “number of” OR “optimal number” OR similar* )

**PEDro**Search 1: Transcranial magnetic stimulation

Search 2: TMS

Search 3: Mapping

Search 4: Motor evoked potential

**NIF**(Transcranial magnetic stimulation OR TMS) AND (MEP OR MEPs OR motor evoked potenti* OR sites OR pulses OR stimuli)

**Scopus**TITLE-ABS-KEY(TMS OR transcranial magnetic stimulation) AND TITLE-ABS-KEY(MEP OR MEPs OR motor evoked potenti* OR sites OR pulses OR stimuli) AND TITLE-ABS-KEY(amplitude OR volume OR CoG OR centre of gravity OR center of gravity OR distance OR shift OR map* OR peak OR cortical reorgani* OR excitab* OR plastic* OR neuroplast* OR cortical represent*) AND TITLE-ABS-KEY(Reliab* OR reproduce* OR valid* OR consisten* OR accura* OR stable OR stability OR agree* OR "number of" OR "optimal number" OR similar*) AND PUBYEAR > 1979

**Web of Science**TOPIC:(Transcranial magnetic stimulation OR TMS) AND TOPIC: (MEP OR MEPs OR motor evoked potenti* OR CMAP OR compound motor action potential OR sites OR pulses OR stimuli) AND TOPIC:(amplitude OR size OR volume OR CoG OR centre of gravity OR center of gravity OR distance OR shift OR peak) AND TOPIC: (Reliab* OR reproduce* OR valid* OR consisten* OR accura* OR stable OR stability OR agree* OR “number of” OR “optimal number” OR similar*)
